# Duodenum and Caecum Microbial Shift Modulates Immune and Antioxidant Response Through Energy Homeostasis in *Hu* Sheep Fed Vegetable Waste and Rice Straw Silage

**DOI:** 10.3390/antiox13121546

**Published:** 2024-12-17

**Authors:** Muhammad Hammad Zafar, Chuang Li, Zhiqi Lu, Yue Lu, Zhenbin Zhang, Ruxin Qi, Usman Nazir, Kailun Yang, Mengzhi Wang

**Affiliations:** 1 College of Animal Science and Technology, Yangzhou University, Yangzhou 225009, China; hammadzafar075@gmail.com (M.H.Z.);; 2 College of Animal Science, Xinjiang Agricultural University, Urumqi 830091, China; 3 State Key Laboratory for Sheep Genetic Improvement and Healthy Production, Xinjiang Academy of Agricultural and Reclamation Science, Shihezi 832000, China

**Keywords:** vegetable waste silage, intestinal microbiota, energy metabolism, antioxidant status immune response, duodenum, caecum

## Abstract

The gradual decline in feed resources for livestock needs alternate ways to ensure non-stop feed supply throughout the year. The objective of this study was to evaluate the impact of vegetable waste and rice straw silage (VTRS) on immune response, antioxidant status, and microbial changes in duodenum and caecum in *Hu* sheep. Eight healthy male *Hu* sheep were randomly distributed into control (fed farm roughage) and VTRS (fed vegetable waste silage) groups for 35 days. Results had shown that silage had less mycotoxin content (*p* < 0.05). The VTRS increased butyrate content in duodenal digesta, while acetate, butyrate, total volatile fatty acids (TVFA), and valerate were enhanced in caecal digesta (*p* < 0.05). The VTRS also increased amylase activity in duodenum and ileum tissues, along with GLUT2 and SGLT1 expressions. In serum, Interleukin-10 (IL-10) concentration and total antioxidant capacity (T-AOC) were increased while malondialdehyde (MDA) was decreased. An increase in T-AOC and GSH-Px activity was also observed, along with increased IL-6, immunoglobulin A (IgA), and catalase in duodenum tissue (*p* < 0.05). *Prevotella* was increased in the duodenum and caecum, with *Prevotellacae UCG-001* and *Christensenellacae R-7 group* representing the VTRS group in the duodenum (*p* < 0.05). KEGG pathway prediction also indicated the enrichment of energy metabolism-related pathways. Significant microbes had shown a significant correlation with immune parameters. It can be concluded that vegetable waste silage has the ability to improve antioxidant status, enhance energy metabolism, and balance intestinal microbiota in *Hu* sheep.

## 1. Introduction

Nutrition is the most important aspect in livestock production as it comprises nearly 70% of the total production cost in all livestock production systems [1]. Until now, farmers try to fulfill animals’ nutritional needs with conventional feed resources; however, continuous genetic improvement in production potential and the development of intensive livestock farming have also raised nutritional standards, which make the farmers think out of the box in order to keep pace with increasing production and demand [2]. Vegetable production in China comprises about 50% of the world’s total production, and China is also the largest producer of Chinese cabbage (≈34.9 million metric tons annually), which is directly linked with a large amount of waste production. This waste contains a significant amount of valuable nutrients as Chinese cabbage is an excellent source of vitamins, minerals, and carbohydrates [3,4,5]. However, moisture is a major constraint for its storage as it can lead to undesirable growth of pathogenic bacteria such as Clostridia, resulting in its spoilage [6]. Apart from that, low DM content in Chinese cabbage was also observed to be associated with low feed intake in goats [7]. To avoid this, it should be mixed with some fibrous ingredients, and rice straw can prove to be a better choice as it contains approximately 85–90% dry matter, and China produces almost 230 million tons of it annually [8]. Rice straw has low digestibility and feed utilization efficiency when it is included in livestock ration [9]. Furthermore, Chinese cabbage waste improved growth performance in goats when used in combination with rice straw compared to its inclusion without rice straw in the ration of goats [7]. So, the use of rice straw along with vegetable waste can not only improve ensiling conditions but also lead to value addition in return. Earlier, several studies have reported the successful use of vegetable byproduct silages in livestock feed, such as broccoli byproducts with wheat straw silage of up to 200 g/kg, which were used to fatten lambs without any negative impact on growth performance, nutrient digestibility, and blood parameters [10]. Beet pulp has also been evaluated with better feed efficiency, better VFA concentration in rumen, and improved meat quality when used in combination with soybean oil in feeding dairy calves [11]. Additionally, Han et al. [12] also reported that replacement of maize silage with mulberry silage had no negative impact on growth performance and rumen fermentation parameters in *Hu* sheep. Moreover, mulberry leaf silage has also improved antioxidant status when it substituted alfalfa silage in Tan lambs [13]. So, vegetable waste can be a good choice.

Numerous studies on rumen microbiome and their link with nutrient metabolism have urged scientists to study microbiomes in other GIT segments as well. Intestinal microbiota represents a highly diverse and heterogeneous ecosystem containing millions of microbes in which predominant phyla are Firmicutes (Gram-positive) and Bacteroidetes (Gram-negative); they were involved in the synthesis of various metabolites, which have the potential to modulate the physiological processes [14]. An interaction of intestinal *Provotellacae* with an overexpression of TLR4 has already been reported as having a significant impact on inflammation modulation in sheep [15]. A few more studies also observed that intestinal microbiota not only participates in nutrient assimilation via secretion of enzymes, which degrade indigestible substances, but also play a crucial role in mediating inflammatory responses and the development of the immune system through the regulation of the gut–brain axis [16,17,18]. A rapid change in pH from the abomasum (2.1–2.3) to the duodenum (around 6), the impact of dietary changes on its microbiota, and the interaction of that microbial change with physiological responses seem to be an interesting aspect that is still unexplored. Moreover, caecum microbiota is thought to perform fermentation of rumen leftovers, producing short-chain fatty acids for energy production, which is essential for the proper functioning of immune cells. However, caecum microbial diversity is relatively less to that in the rumen [19]. The difference in microbial abundance between the rumen and the caecum has already been reported in various studies [20,21,22]. However, the functional potential of caecum microbiota is still to be explored for a better understanding of hindgut microbiota.

We hypothesized that vegetable waste and rice straw silage may alter intestinal microflora, which can result in immune response modulation. The objective of this study was to investigate the role of vegetable waste silage in enhancing the health status of Hu sheep and to provide a scientific foundation for its dietary application.

## 2. Materials and Methods

### 2.1. Silage Preparation and Toxin Content Estimation

Vegetable waste (Chinese cabbage waste) and rice straw were taken from Suqian City, Jiangsu Province, China. *Lactobacillus plantarum* and cellulase, being critical components in the silage-making process, were acquired from Guangzhou Greenfield Biotechnology Co., Ltd., Guangzhou, China. For ensiling, both rice straw and vegetable waste were chopped to 2–3 cm lengths and mixed at 4:6 ratio (*w*/*w*) with subsequent addition of *Lactobacillus plantarum* and cellulase at 0.035 g/kg and 0.25 g/kg, respectively. That mixed composite was then vacuum-packed using Meggis vacuum machine, which was later sealed and stored in silage bales for 45 days in cool environment to facilitate anaerobic fermentation. Total of 51 tons of silage was prepared, with each wrap having 300 kg in it. This incubation was then followed by a silage sample collection, for which a composite sampling technique was adopted; samples from 5 to 6 random bales were collected. This is followed by mixing these to obtain a representative sample. This process was repeated 3 times to get replications for further analysis. Chemical composition of major components of vegetable waste silage and prepared silage is given in Table 1 and Table 2. Toxin contents of both experimental diets, including aflatoxins, ochratoxin A, and zearalenone, were estimated using ELISA kits from Shanghai Amperexamination Technology Co., Shanghai, China. Contents of vitamin A (V_A_), vitamin B_2_ (V_B2_), V_C_, and vitamin E (V_E_) were determined by commercial kits acquired from Shanghai Enzyme Link Biotechnology Co., Ltd. (Shanghai, China).

### 2.2. Animal Model and Experimental Design:

Eight male *Hu* sheep (5.5 months old) having an average initial weight of 39.45 ± 4.62 kg with good health status were randomly divided into two dietary treatments (Control & VTRS) containing 4 animals per treatment. Control group was fed a conventional sheep farm roughage based on peanut seedlings, maize husk, and sorghum hulls. However, VTRS group was offered vegetable waste silage as primary and sole roughage source. Forage-to-concentrate ratio was 50:50 on dry matter basis. Feed formulation was performed according to the standards of the National Research Council (NRC, 2007) concerning the nutritional requirements of meat sheep (40 kg body weight; 400 g daily gain) (Table 3).

**Figure 1:**
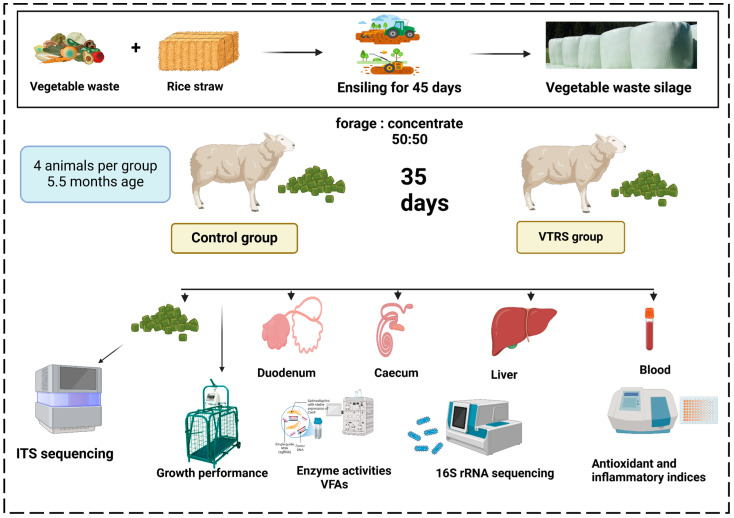
Schematic diagram showing outline of experiment.

The total feeding period lasted for 5 weeks, including 1st week of the pre-feeding period, while the rest of the duration was the collection/feeding period. All animals were housed in clean individual pens and went through standardized deworming and immunization protocols prior to start of feeding period. Each sheep received two daily feedings (8:00 and 18:00), and ad lib access to feed and clean drinking water was given throughout pre-feeding and feeding periods. Figure 1 shows outline of experimental plan.

### 2.3. Determination of Growth Performance

At the start of the feeding period, the fasting weight of each sheep was recorded prior to the 1st morning feeding and termed as the initial weight (IW), while the final weight (FW) was recorded after the completion of the feeding period before next morning’s feeding to calculate average daily gain (ADG). Feed offered and residual feed for each sheep were recorded throughout experimental duration to calculate average daily feed intake (ADFI). Ultimately, the feed-to-weight ratio (F/W) was derived based on the calculated values of ADG and ADFI.

Average daily gain (ADG) = Final weight − Initial weight/28

Average daily feed intake (ADFI) = (Feed offered × Dry matter content) − (Residual feed × Residual feed dry matter content)

Feed/gain = ADFI/ADG.

### 2.4. Sample Collection

At the end of the feeding experiment, sheep were fasted for 24 h with an ad lib supply of drinking water before slaughtering. Then, slaughtering was performed after blood sampling from jugular vein and bloodletting. Afterward, the duodenum and caecum were carefully cut from their middle part to collect the digesta (5 mL) in 2 sterile tubes from each sheep for VFA determination. For tissue samples, digesta from duodenum, jejunum, ileum, and caecum was completely removed, all the segments were rinsed with saline, and then clean tissues were taken (≈15 cm for small intestinal segments; 2 × 2 cm, 3 cm length for caecum) and stored for determination of digestive enzyme activities, cytokines, immunoglobulins, and antioxidant parameters. Meanwhile, liver tissue samples were also collected during slaughtering for measurement of antioxidant and immune indices. All samples were collected in cryovials, immediately kept in liquid nitrogen on the farm, and later shifted to a lab and stored at −80 °C for further use.

### 2.5. Determination of Digestive Enzyme Activities and VFA Content

Approximately 0.1 g of tissue samples from the duodenum, jejunum, and ileum were taken and subsequently placed into 1.5 mL sterile enzyme-free centrifuge tubes. These tissues were mixed with physiological saline at a ratio of 1:9 followed by grinding through tissue grinder and centrifugation at 12,000 rpm for 10 min. Then, supernatant was immediately taken to measure activity of amylase, lipase, and trypsin. Above parameters were quantified using commercial kits purchased from Nanjing Jiancheng Biotechnology Co., Ltd., while VFA content in digesta was estimated through gas chromatography (GC-14B, Kyoto, Japan) along with meteorological chromatography internal standard method; see Pan et al. [23]. Meanwhile, TVFA and A:P ratio were computed. Apparatus and operational conditions for gas chromatography were similar, as described by Zhang et al. [24].

### 2.6. Determination of Antioxidant and Inflammatory Parameters

Concentrations of total antioxidant capacity (T-AOC), glutathione peroxidase (GSH-Px), superoxide dismutase (SOD), malondialdehyde (MDA), and catalase (CAT) in serum and tissue samples of liver, duodenum, and caecum were determined using assay kits purchased from Nanjing Jiancheng Biotechnology Co., Ltd., Nanjing, China. Moreover, inflammation indices such as IL-1β, IL-6, IL-8, IL-10, TNFα, IgA, and IgM were determined using ELISA kits for sheep purchased from Beijing Solarbio Science & Technology Co., Ltd., Beijing, China. The analysis was performed strictly following the instructions of the manufacturer.

### 2.7. Determination of Gene Expression of Glucose and Amino Acid Transporters

The total RNA of each intestinal tissue was extracted using the TaKaRa RNAios Plus (Total RNA Extraction Reagent) kit. The RNA concentration and quality were determined prior to reverse transcription in order to synthesize cDNA (PrimeScript RT reaction kit with gDNA Eraser, TaKaRa). Specific primer sequences of glucose and amino acid transporter-related genes are shown in Table 4, while β-actin was used as a reference gene. Relative expression of related genes was detected using the TB Green Primer Ex Taq (TaKaRa) kit, while the reaction system used was 10 μL.

### 2.8. Microbial DNA Extraction and 16 S/ITS rRNA Sequencing

Genomic DNA from feed, duodenum, and caecum samples was extracted using HiPure Soil DNA Kit (D3142B, Magen, Guangzhou, China). Meanwhile, quality and quantity evaluation of extracted DNA was conducted through Nanodrop 2000 spectrophotometer (Thermo Fisher Scientific, Wilmington, DE, USA). Characterization of microbial populations was carried out through bacterial 16S rRNA and fungal ITS rRNA genes. Amplification of V3–V4 region of 16S rRNA gene was performed using the GeneAmp^®^ 9700 PCR apparatus (ABI, Los Angeles, CA, USA) with the primers 341F (5′CCTACGGGNGGCWGCAG-3′) and 806R (5′GGACTACHVGGGTWTCTAAT-3′), while ITS2 region of ITS rRNA genes was amplified using primers ITS3F (5′-GCATCGATGAAGAACGCAGC-3′) and ITS4R (5′-TCCTCCGCTTATTGATATGC-3′). Ultimately, resultant amplified fragments were subjected to sequencing on the Illumina Novaseq PE250 sequencing platform (Genepioneer Biotechnologies Co., Ltd., Nanjing, China) in accordance with the specific protocols described by Zhang et al. [25].

### 2.9. Data Analysis

Normal distribution and homogeneity of data were tested using IBM SPSS Statistics V26.0 (Chicago, Illinois, USA). Means comparison of toxin content, antioxidant and inflammatory indices, digestive enzyme activity and gene expression of nutrient transporters was carried out through independent samples *t*-test. Species richness (Chao1 index) and diversity (Simpson and Shannon indices) were calculated in R using picante package, while differences among groups were evaluated by non-parametric Mann–Whitney U test with IBM SPSS Statistics V26.0. Venn diagrams and Principal coordinate analysis (PCoA) diagrams were drawn through the ggplot2 package of the R language. Only those bacterial taxa with an abundance > 0.1% in at least one sample were analyzed. KEGG microbial gene function prediction analysis was performed using PICRUSt according to previous protocol described by Chuang et al. [26]. Redundancy Analysis (RDA) was performed to evaluate the interaction between microbiota and VFAs of respective segments using a vegan package in R language. Spearman correlation network was drawn to show the associations between significant microbial communities of duodenum and caecum with antioxidant and inflammatory indices using R language and Gephi software (version 0.10). Threshold for correlation was set at *p* < 0.05; r > 0.75 and <−0.75.

## 3. Results

### 3.1. Toxin Content in the Feed

Toxin content showed significant differences between control and VTRS (*p* < 0.05). Toxin concentrations in both diets were within safety standards; however, on treatment comparison, ochratoxin A and zearalenon contents were significantly lower in VTRS compared with the control diet (*p* < 0.05) (Table 5). Vitamin content also showed significant differences among treatments as V_A_, riboflavin, and Vc had shown a significant increase in the VTRS group compared with control (*p* < 0.05) (Table 5).

### 3.2. Fungal Microbial Structure of Experimental Diets

**Figure 2:**
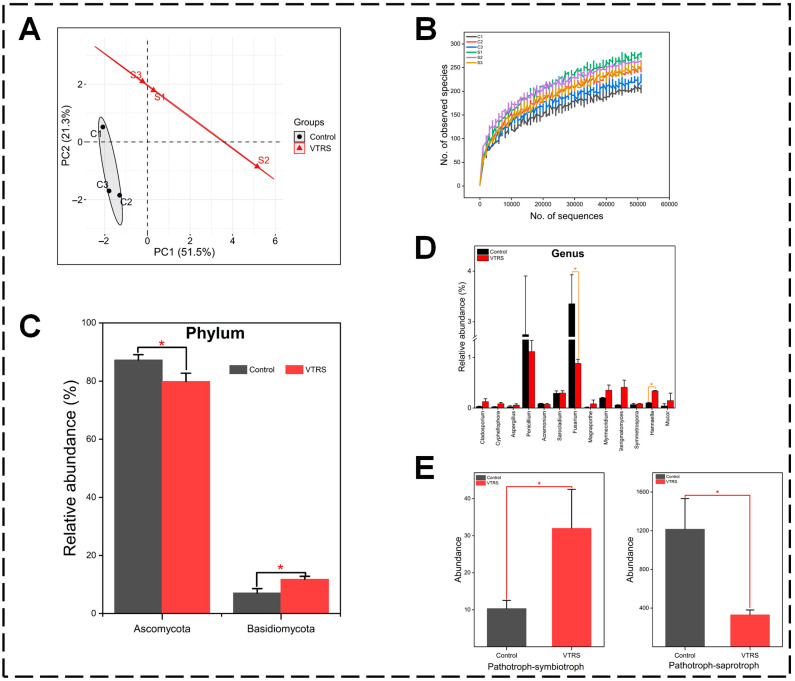
Fungal microbial structure in experimental diets: (**A**) PCoA plot showing beta diversity. (**B**) Species accumulation curve. (**C**) Comparison of top two phyla among groups. (**D**) Differential analysis of microbial communities at genus level. (**E**) Functional enrichment analysis showing differences among the groups. “*” on the bars shows significant difference among the groups.

Upon ITS rRNA sequencing, a total of 74,639 and 64,961 clean tags were obtained after quality check in control and VTRS, respectively, while on the basis of effective tags and OTUs clustering (97% similarity index), 253 and 282 OTUs on average were derived for both control and VTRS group respectively. Meanwhile, the number of OTUs showed a significant spike in the silage diet (Table S2). Among alpha diversity indices, the Chao1 index showed an increase in the VTRS group, while other indices remained non-significant (*p* > 0.05) among both dietary treatments (Table S1). The species accumulation curve had reached a plateau at around 250 observed species in VTRS samples and 210 in control samples; however, the trend and distribution of species across samples were uniform (Figure 2B).

At the phylum level, Ascomycota and Basidiomycota are the two main phyla in both control and silage diets, which comprised about 85% and 5% of total phyla. Both of them showed the opposite trend as Ascomycota was significantly reduced, and Basidiomycota was increased in silage diets compared with the control diet (*p* < 0.05) (Figure 2C). At the genus level, VTRS had shown a significant reduction (*p* < 0.05) in *Fusarium* and an increase in *Hannaela* abundance, while the highest abundance was observed for *Penicilium*, which remained non-significant (*p* < 0.05) across treatments (Figure 2D). Functional prediction revealed that the majority of functions (>40%) were classified as saprotrophs, while in VTRS, plant pathotroph–saprotroph function was reduced (*p* < 0.05), and pathotroph–symbiotroph function was increased (Figure 2E).

### 3.3. Growth Performance

Growth performance was evaluated on the basis of average daily weight gain (ADWG), average daily feed intake (ADFI), and the feed-to-gain (F/G) ratio, and all of them showed no significant differences (*p* > 0.05) among dietary groups (Table 6).

### 3.4. Digestive Enzymes Activity, VFA Content and Nutrient Transporters

Table 7 indicates that animals fed VTRS had been observed with increased amylase activity (*p* < 0.05) in the duodenum and ileum tissues. However, lipase and trypsin activity remained unaffected (*p* > 0.05) in all segments of the small intestine across both dietary treatments.

Regarding VFA content in the duodenum, all VFAs had shown non-significant differences (*p >* 0.05) between the two dietary groups; however, butyrate and TVFA concentration were increased (*p* < 0.05) in VTRS group. Acetate, Isobutyrate, valerate, and isobutyrate molar proportions were decreased in the VTRS group, while a significant increase in the molar proportion of butyrate was seen in the VTRS group (Table 8).

In the caecum, the majority of VFAs, including acetate, butyrate, valerate, and TVFA, were increased (*p >* 0.05) in VTRS-fed animals. For molar proportions, butyrate underwent a significant increase in the VTRS group along with a slight enhancement in valerate proportion (Table 9).

Gene expression of various glucose and amino acid transporters in various segments of the small intestine has been determined which had shown no significant differences (*p >* 0.05) in duodenum and jejunum, while, in ileum tissue, vegetable waste VTRS feeding increased (*p* < 0.05) the expression of two glucose transporters, i.e., GLUT2 and SGLT1 (Table 10).

### 3.5. Bacterial Microbial Community and Functional Prediction Enrichment in Duodenum

A total of 58553 clean tags in control and 64563 in VTRS were obtained after quality check from raw tags derived from 16S rRNA sequencing. We have also noted no significant differences in the number of OTUs among dietary groups (Table S2).

**Figure 3:**
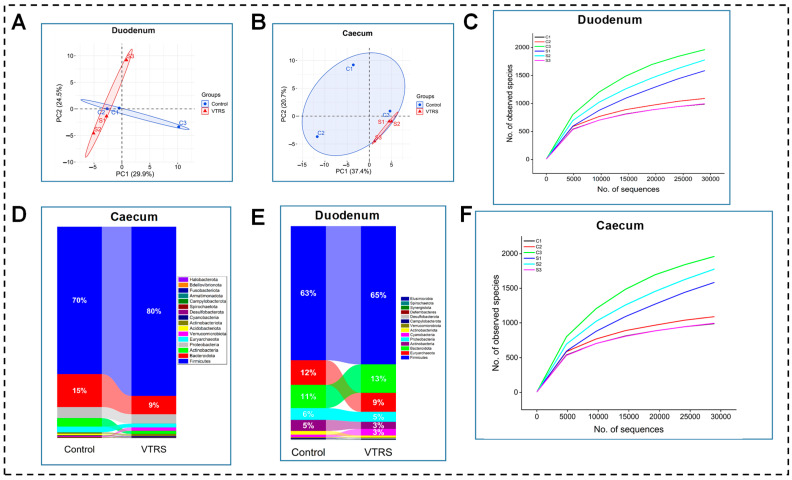
Bacterial diversity and phylum comparison among dietary groups in duodenum and caecum. (**A**) PCoA plot showing beta diversity for microbiota in duodenum. (**B**) PCoA plot showing beta diversity for microbiota in caecum. (**C**) Species accumulation curve showing distribution of species in duodenum. (**D**) Comparison of most abundant phyla in duodenum. (**E**) Comparison of most abundant phyla in caecum. (**F**) Species accumulation curve showing distribution of species in caecum.

In SAC, curves for samples from both the groups exhibited a similar pattern with more steepness at the start and showed that species abundance was higher for dominant species, which gradually became flat as the occurrence of rare species. It can also be seen that after the detection of 500 species, abundance becomes very low and eventually becomes flat, which reveals no new species detection after a certain amount of testing (Figure 3C). Venn diagram showed 1935 unique OTUs in the VTRS group, and the control group showed 1200 unique OTUs; however, 1158 OTUs were shared among the groups (Figure S1A). No alpha diversity indices exhibited any significant difference (*p >* 0.05) among the dietary groups (Table S3).

**Figure 4:**
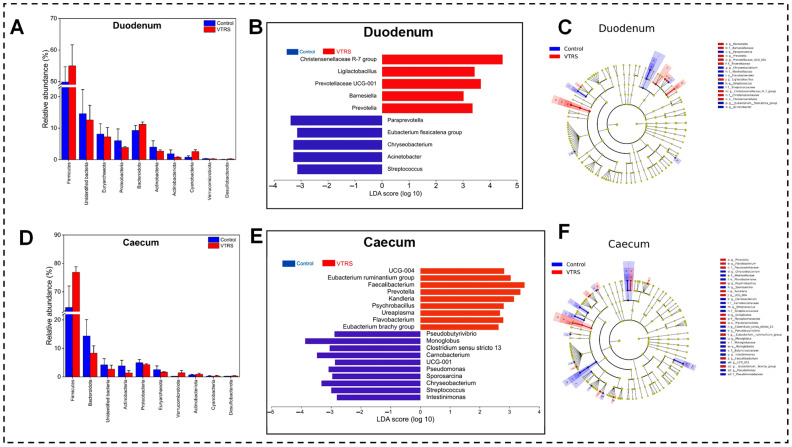
Differences in microbial communities of duodenum and caecum: (**A**) Differential analysis of microbial communities at phylum level in duodenum. (**B**) LeFSe analysis showing biomarkers at genus level in duodenum. (**C**) Cladogram showing differences at all taxonomic levels among the groups. (**D**) Differential analysis of microbial communities at phylum level in duodenum. (**E**) LeFSe analysis showing biomarkers at genus level in caecum. (**F**) Cladogram showing differences at all taxonomic levels among the groups in caecum.

**Figure 5:**
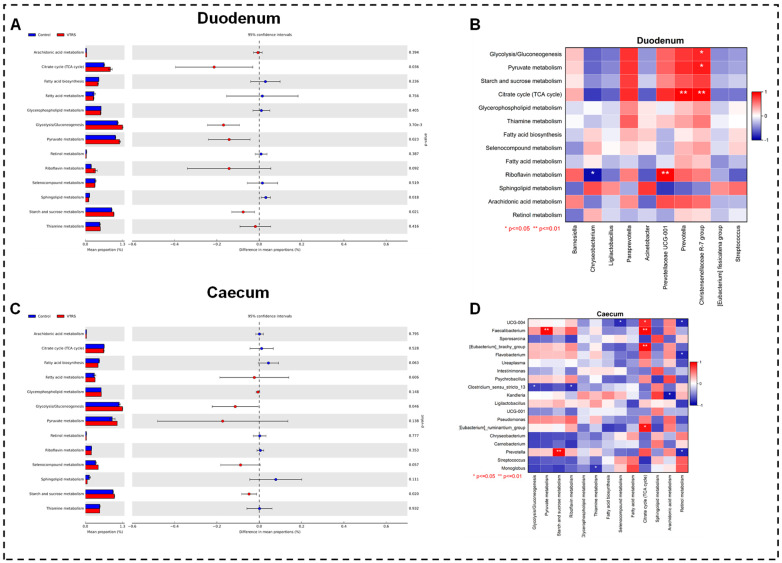
Differences in metabolic pathways enrichment among dietary treatments and correlation analysis between significant microbial communities and metabolic pathways: (**A**) Differential analysis of metabolic pathways in duodenum. (**B**) Correlation between significant microbial communities and metabolic pathways in duodenum. (**C**) Differential analysis of metabolic pathways in caecum. (**D**) Correlation between significant microbial communities and metabolic pathways in caecum.

At phylum level, both groups followed a similar trend for control and VTRS treatments because the top three phyla, which are Firmicutes, Bacteriodetes, and Euryarchaeota, comprised 63% and 65%, 11% and 13%,and 12% and 9%, respectively. However, Bacteriodota was the second most abundant phylum in VTRS treatment and control treatment had Euryarchaeota at the second rank (Figure 3E). Comparing abundances between the groups, we found that no phyla had shown any significant difference (*p >* 0.05) (Figure 4A). At the genus level, distinct genera with significant differences between control and VTRS groups have been observed because *Paraprevotella*, *Christensenellacae R-7 group*, *Prevotellacae UCG-001*, *Barnesiella* and *Prevotella* were significantly more abundant (*p* < 0.05) in the VTRS group, while *Ligilactobacillus*, *Eubacterium fissicatena*, *Chryseobacterium*, *Acinetobacter,* and *Streptococcus* had been observed with comparatively lesser abundance (*p* < 0.05) in VTRS group (Figure 4B). Figure 4C revealed no significant changes in higher taxonomic levels as in the VTRS group. The greatest change was seen on the order level in Christenellales; however, the control group exhibits a change in the highest taxonomic level at order Flavobacteriales. Functional prediction of metabolic pathways had shown that VTRS feeding significantly improved (*p* < 0.05) the enrichment of energy metabolism-related pathways such as starch and sucrose metabolism, glycolysis, citrate cycle, and pyruvate metabolism (Figure 5A).

### 3.6. Bacterial Microbial Community and Functional Prediction Enrichment in Caecum

A total of 67838 in control and 67304 clean tags in the VTRS group were derived from 16S rRNA sequencing after a quality check. From effective tags, 1145 and 1533 OTUs were obtained based on clustering at 97% similarity (Table S2). Around 1103 OTUs were shared between the two groups, while 1817 and 540 were unique OTUs in control and VTRS, respectively (Figure S1B). The SAC had shown similar trends for both treatments, having steepness at the start and reaching a plateau after certain sequences with uniform distribution (Figure 3F). Alpha diversity, i.e., Shannon, Simpson, ACE, and Chao1 had shown no significant differences (*p* > 0.05) across treatments (Table S3).

At the phylum level, both groups had the same phyla at the top 3 positions, with Firmicutes being the most abundant, followed by Bacteroidetes and Proteobacteria. However, in the control treatment, Actinobacteria replaced Euryarchaota at the fourth spot in abundance (Figure 3D). Regarding the difference between the dietary groups, Verrucomicrobiota and Acidobacteriota showed a significant increase (*p* < 0.05) in relative abundance in animals fed VTRS compared with the control group (Figure 4D). At the genus level, *Romboutsia* is the most abundant genera in both groups, followed by *Paeniclostridium*, while *Christensenellacae R-7 group* is the third most abundant in the VTRS and *Saccharofermentans* in the control group. *Erysipelatoclostridiacace UCG-004*, *Eubacterium ruminantium group*, *Faecalibacterium*, *Prevotella*, *Pseudomonas*, *Psychrobacillus*, *Ureaplasma*, *Flavobacterium,* and *Eubacterium brachy group* represented VTRS group and *Pseudobutyrivibrio*, *Monoglobus*, *Clostridium sensu stricto 13*, *Carnobacterium*, *Ruminococcacae UCG-001*, *Kandleria*, *Sporosarcina*, *Chryseobacterium*, *Streptococcus* and *Intestinimonas* represented control group (Figure 4E). Figure 4F showed no significant difference above the order level as Monoglobales and Flavobacteriales were the only two orders representing the control group. Data from the KEGG pathway prediction suggest that some of the metabolic pathways were observed as having significantly different regulation across the groups. The VTRS upregulated amino acid and nucleotide sugar metabolism, glycolysis/gluconeogenesis, starch and sucrose metabolism, and lipoic acid metabolism while downregulated lipid metabolism in the control group.

### 3.7. Antioxidant and Inflammatory Indices

Antioxidant and inflammatory indices had shown significant results in different sites, such as in serum; VTRS silage feeding increased total antioxidant capacity and decreased MDA content (*p* < 0.05), while all other indices were statistically the same across the treatments. In the liver, only MDA content was significantly reduced in the VTRS group. However, a non-significant trend (*p >* 0.05) has been exhibited by all other indices (Table 11).

Among GIT segments, and in the duodenum, IL-6 and IgA had shown a significant rise (*p* < 0.05) in animals fed VTRS. From an antioxidant perspective, T-AOC, SOD, and GSH-Px had also shown increased activity (*p* < 0.05) in the VTRS group from the duodenum. The caecum had also been observed with significant changes in antioxidant and inflammatory indices as T-AOC, GSH-Px, and IL-10 had shown a significant increase (*p* < 0.05) in the VTRS group compared with the control group (Table 12).

### 3.8. Interaction of Microbes and Physiological Indices

Correlation analysis was performed to evaluate the interactions between various parameters. In the duodenum, *Prevotella* had shown a strong positive correlation with the citrate cycle, and the *Christensenellacae R-7 group* exhibits positive correlations with glycolysis/gluconeogenesis and pyruvate metabolism. Moreover, a significant negative correlation was observed between *Chryseobacterium* and riboflavin metabolism (Figure 5B). In the caecum, *Faecalibacterium* was positively correlated with the TCA cycle pathway and with starch and sucrose metabolism, while *Prevotella* also showed a strong positive correlation with starch and sucrose metabolism (Figure 5D).

**Figure 6:**
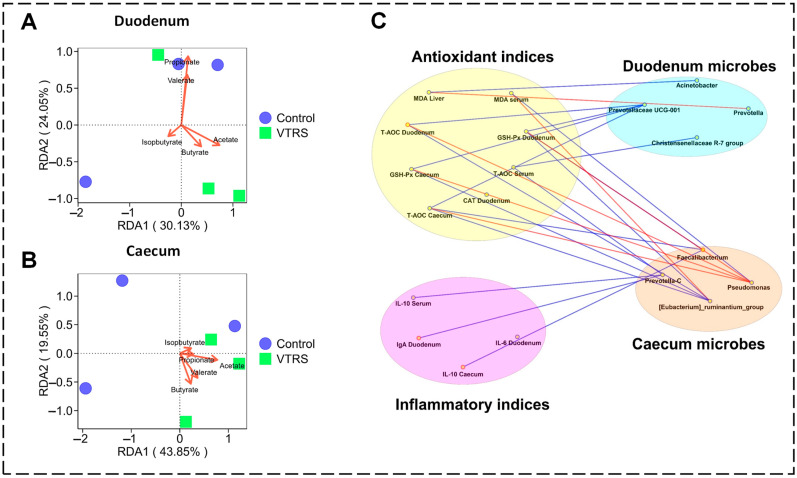
Redundancy analysis (RDA) and correlation network: (**A**) RDA analysis showing association between duodenal microbial communities and VFAs; (**B**) RDA analysis showing association between cecal microbial communities and VFAs; (**C**) Spearman correlation network between significant intestinal microbes and significant immune indices. Blue lines show significant positive correlations (*p* < 0.05; r > 0.75), and red lines represent significant negative correlations (*p* < 0.05; r < −0.75).

Redundancy analysis (RDA) in the duodenum had shown that the correlation sequence is acetate > butyrate > isovalerate > valerate > propionate with VTRS samples (Figure 6A), while, in caecum samples, the correlation strength with VTRS group microbial communities from stronger to weaker is butyrate > acetate > valerate > propionate > isobutyrate (Figure 6B).

Figure 6C shows the correlations between significant intestinal microbes and immune parameters in which significant microbes in duodenum samples of the VTRS group were mainly associated with antioxidant status and caecum microbes exhibiting their interaction with inflammatory parameters. *Prevotellacae UCG-001* shows a positive correlation with CAT, GSH-Px, and T-AOC from systemic and gut origin, while *Prevotella* represents a negative correlation with MDA in the liver. The *Christensenellacae R-7 group* also shows a positive association with T-AOC in serum, while among caecum microbes, *Prevotella* and *Faecalibacterium* had shown positive interaction with the anti-inflammatory cytokine IL-10.

## 4. Discussion

Mycotoxin contamination represents critical silage-associated limitation due to fungal activity that has adverse effects on palatability, production performance, immune health, and overall feed efficiency [27]. As the high moisture content of vegetables makes them vulnerable to mold growth during ensiling, we have used rice straw for providing better ensiling conditions, which resulted in relatively low toxin content (Ochratoxin A and zearalenon) in VTRS along with decreased *Fusarium* abundance as it is the major producer of zearalenone in many plants and crops in different parts of the world [28,29,30]. The zearalenone and its metabolites (α-zearalenol and β-zearalenol) interact with cytochrome P450 enzymes that inhibit CYP3A4 and CYP1A2, leading to endocrine disruption and immunosuppressive effects [31]. Furthermore, it can also inhibit the TLR2/NFκB signaling pathway, leading to reduced innate immune response and susceptibility to infections [32]. Similar to our findings, Das et al. [33] reported a reduction in moisture content and better silage quality when vegetable waste was ensiled with rice straw silage, which resulted in better production performance and health status in lambs. Furthermore, Chinese cabbage, a major vegetable portion of the VTRS group, is reported to be an excellent source of vitamins A, C, and K [34], which is similar to our study as we also observed higher V_A_, V_C_, and riboflavin content in VTRS diet compared with control.

In the present study, we have observed microbial shifts in duodenum and caecum at the genus level. However, no significant differences were seen at a higher taxonomic level. This indicates the integrity of the core microbial structure, which is necessary for various physiological functions. However, the *Christensenellaceae R-7 group*, *Ligilactobacillus*, and *Prevotella* were increased in the VTRS group, which are recognized for their roles in fermenting carbohydrates. *Prevotella* possesses specialized gene clusters termed as polysaccharide utilization loci, which encode enzymes involved in carbohydrate breakdown [35]. Some of the *Prevotella* strains also produce α-amylase needed for the breakdown of starch [36]. The *Christensenellacae R-7 group* is also reported to have a role in nutrient absorption and maintaining energy balance, being a part of the microbiome cluster in goats exhibiting high energy efficiency and butyrate production [37]. In line with these microbial shifts, butyrate content in duodenal digesta was also increased in the VTRS group and showed microbial contribution in improving intestinal health as butyrate serves as an energy source for intestinal epithelial cells, which enhances tight junction integrity and also exerts anti-inflammatory effects [38]. Ligilactobacillus, known for its probiotic characteristics, contributes to mucosal immunity by promoting the production of immunoglobulin A (IgA), which we also have recorded in this study. Similarly, in the caecum, the predominance of *Prevotella*, *Eubacterium ruminantium*, and *Faecalibacterium* further highlights the superior fermentation capacity of VTRS group as these microbes are involved in carbohydrate digestion. *Faecalibacterium*, an eminent butyrate producer, plays a critical role in anti-inflammatory mechanism which aligns with which with elevated cecal levels of IL-10 and GSH-Px activity within the caecum in the VTRS group. These microbial changes along with increased concentration of VFAs in duodenum and cecal digesta was further supported by KEGG pathways prediction in which we have observed energy related pathways i.e., glycolysis and TCA cycle relatively more enriched in VTRS group as these pathways enhance energy extraction from feed, contributing to energy homeostasis.

Different nutrients such as carbohydrates, fats, and proteins are degraded by digestive enzymes in the intestine to produce free amino acids, glucose, fructose, etc. We have measured the activity of digestive enzymes in small intestinal tissue and observed an increase in amylase activity in duodenum and ileum tissues. This increase can be attributed to the higher availability of substrate from VTRS as it contains vegetables as a carbohydrate source and rice straw as a fiber source, which indicates enhanced nutrient assimilation and absorption efficiency. An increase in amylase activity indicates more breakdown of carbohydrates in the intestine, whose final product is glucose. To check the absorption efficiency of the intestine, we measured the expression of glucose and amino acid transporters and recorded higher expression of GLUT2 and SGLT1 in the VTRS group. GLUT2 and SGLT1 are key regulators of glucose absorption in intestinal tissue, and an increase in their expression is an indication of increased glucose absorption from the intestinal lumen to systemic circulation [39,40]. Along with this phenomenon, we have observed non-significant differences in ADG among both dietary groups. Contrary to these findings, Das et al. [33] replaced maize silage with vegetable waste and rice straw silage and recorded increases in ADG without any significant change in initial and final weight. Differences in results in these two experiments can be due to the duration as they performed feeding trials for 90 days compared with our study (35 days). Another reason could be the initial weight. We used animals with relatively higher body weight (≈39 kg) than the other animals (≈10 kg). Moreover, number of animals was also a limitation in determining growth performance. So, these things should be kept in mind for the next time while conducting the experiment on non-conventional feed source for growth determination.

The proper balance of inflammatory cytokines and antioxidants is critical for optimum health status in livestock as inflammatory cytokines are immune response mediators, whereas antioxidants mitigate oxidative stress [41,42]. Anti-inflammatory cytokines (IL-10) are critical in finishing neuro-inflammatory processes, whereas pro-inflammatory cytokines (IL-1, IL-6, TNF-α) are a critical part of acute inflammatory response [43,44]. In our study, we have observed an increase in IL-10 concentration caecum along with an increase in IgA in the duodenum. This improvement in immune response locally in the gut can be due to the microbial shift within respective segments as *Ligilactobacillus*, abundant in VTRS, has been reported for having an association with IgA production, while Faecalibacterium, possessing anti-inflammatory properties, is already being used for the development of next-generation probiotics [45,46]. Along with inflammatory mediators, VTRS improved antioxidant status both in the intestine and systemic circulation as T-AOC was improved in the serum, duodenum, and caecum. Meanwhile, MDA content, an indicator for lipid peroxidation, in the liver and serum was reduced in the VTRS group. Reduction in serum and liver MDA may be due to higher vitamin C content in vegetable waste silage. Vitamin C has the ability to reduce lipid peroxidation by direct scavenging, regeneration of other antioxidants such as vitamin E, disrupting chain reactions of lipid peroxidation in cell membranes and lipoproteins and supporting other antioxidant enzymes such as CAT and GSH-Px, which results in lowering ROS and ultimately lipid peroxidation. Similar to our findings, Ishida et al. [47] also observed improved antioxidant status and rumen fermentation without any change in feed intake and weight gain in sheep-fed winery waste. Similarly, Mulberry leaf silage also improved antioxidant enzyme activities in serum and reduction in serum MDA content while replacing alfalfa silage without affecting growth performance [13].

Improvement in energy homeostasis through catabolic reactions helps in cellular adaptation to stress and immunity regulation through the physiological production of mitochondrial ROS up to a certain level, which is confirmed in this study through inflammatory response [48]. Similar to this study, the application of Chinese herbs enhanced antioxidant status at cellular and mitochondrial level by stimulation in ATP synthesis via intermediatory reactive oxidant species [49]. Moreover, Fu et al. [50] observed that glucose metabolism in interaction with pyruvate carboxylase helps in the synthesis of glutathione, which is an ROS scavenger in many physiological and pathological conditions and observed in relatively higher concentrations in the duodenum of the VTRS group. However, contrary to our observations, the hyperactivity of energy metabolism sometimes causes overproduction of ROS, which can adversely affect antioxidant status. Therefore, the extent of that increase in energy metabolism is still not fully explored. So, exploring the mechanisms involved in enhancing immune and antioxidant status along with improved energy metabolism upon VTRS feeding needs further experimentation.

## 5. Conclusions

The inclusion of VTRS increased VFA content in the duodenum and caecum and enhanced amylase activity along with increased glucose absorption in small intestinal tissue. It also improved antioxidant enzyme activity locally in the gut and reduced lipid peroxidation in the serum and liver. Additionally, VTRS also increased the abundance of useful bacteria such as *Prevotella*, *Christensenellacace R-7 group*, and *Ligilactobacillus* in the duodenum but also enhanced VFA-producing *Faecalibacterium* in the caecum. Our results suggest that VTRS inclusion might improve the health status of Hu sheep by improving antioxidant status and immune response, enhancing energy metabolism, and promoting balanced intestinal microbiota. Further research could deeply explore the mechanism of VTRS improving energy metabolism, its beneficial extent for better health status, and microbial interaction with immunity in *Hu* sheep.

## Figures and Tables

**Table 1:** Chemical composition of major components of vegetable waste silage.

Items (%)	Chinese Cabbage Waste	Rice Straw
Dry matter (DM)	4.13	79.52
DM basis (%)		
Crude protein (CP)	19.97	5.49
Ash	11.55	9.97
Neutral detergent fiber (NDF)	19.41	66.73
Acid detergent fiber (ADF)	11.97	39.42
Water Soluble carbohydrates	7.05	4.84
The above values are measured values		

**Table 2:** Chemical composition of vegetable waste silage.

Items (%)	Vegetable Waste Silage
Dry matter (DM)	24.97
DM basis (%)	
Crude protein (CP)	7.50
Ash	9.84
Neutral detergent fiber (NDF)	51.79
Acid detergent fiber (ADF)	31.56
Water Soluble carbohydrates	4.37
The above values are measured values	

**Table 3:** Ingredient and nutrient composition of experimental diets.

Ingredients	Control	VTRS
Peanut seedlings	30.00	–
Maize husk	15.00	–
Sorghum hulls	5.00	–
Vegetable waste silage	--	50.00
Maize grains	34.00	34.00
Soybean meal	7.00	5.50
Wheat Bran	7.50	8.00
Maize gluten	–	1.00
NaHCO3	0.50	0.50
Premix contained	0.50	0.50
Salt	0.50	0.50
Total	100.00	100.00
Metabolizable energy (MJ/Kg)	13.42	14.63
CP (%)	15.08	15.11
Ash (%)	4.36	12.33
NDF (%)	47.64	48.23
ADF (%)	23.71	27.17
Ca (%)	0.48	0.45

An amount of 1 kg of premix composed of Vitamin A 130 klU, Vitamin D 30 kRJ, Vitamin E 130 klU, Fe 0.8 g, Mn 1.0 g, Zn 3.0 g, Cu 0.2 g, Se 8 mg, Ca 8 g, P 1 g, and NaCI 100 g. All values are on DM basis.

**Table 4:** Primer sequences for genes related to glucose and amino acid transporters.

Genes	Accession Numbers	Forward Primer (5′–3′)	Reverse Primer (3′–5′)
^1^ GLUT2/SLC2A2	AJ318925.1	TGTTTCACTGGATGACGGAAT	AGCCCAAGAGACTGGTGTTG
^2^ SGLT1/SLC5A1	NM_001009404	GTGCAGTCAGCACAAAGTGG	CCCGGTTCCATAGGCAAACT
^3^ SLC38A2	XM_004006421	TTCATTTGTCTGCCATCC	TCAGGTATCCAAAGAGGG
^4^ SLC3A2	XM_060404045	AGAACATCACTAAGAGCGTCAG	AACAGGTCCTTGGTGGGT
^5^ SLC6A19	XM_027980192	TCGGTCATCGTGTCTGTGAT	GGAAGCAGTCGTCGTAGCG
^6^ SLC1A5	XM_027978525	GGGGCGAGGTTGAGGGTAT	TGAAGGAGTTGAAGAAGCGAAT
β-actin	NM_001009784.3	AGCCTTCCTTCCTGGGCATGGA	GGACAGCACCGTGTTGGCGTAGA

^1^ Glucose transporter 2; ^2^ Sodium–glucose cotransporter 1; ^3^ Amino acid transporter 2; ^4^ Solute carrier family 3 Member 2; ^5^ Solute carrier family 6 members 19; ^6^ Solute carrier family 1 member 5.

**Table 5:** Toxin contents in experimental diets.

Items	Groups		SEM	*p*-Value
	Control	VTRS		
Aflatoxin (ppb)	3.7	4.1	0.14	0.17
Ochratoxin A (ppb)	15.8 ^a^	11 ^b^	0.45	<0.01
Zearalenon (ppb)	89.9 ^a^	28.4 ^b^	0.4	<0.01
V_A_ (μg/L)	71.9 ^b^	75.9 ^a^	0.85	0.032
V_B2_ (μg/L)	3.5 ^b^	4.8 ^a^	0.09	0.015
V_C_ (μg/L)	22.9 ^b^	28.1 ^a^	0.35	<0.01
V_E_ (μg/L)	8.1	7.6	0.2	0.11

SEM—standard error of mean; means within a row showing different superscripts were significant among treatments (*p* < 0.05); all values are on DM basis.

**Table 6:** Effect of VTRS on growth performance (n = 4).

Items	Groups		SEM	*p*-Value
	Control	VTRS		
Average daily feed intake (g)	1157.5	1117.5	40.52	0.529
Average daily weight gain (g)	220.1	247.5	27.05	0.501
Feed/gain ratio	5.392	4.682	0.448	0.307

SEM—standard error of mean. Means within a row showing different superscripts were significant among treatments (*p* < 0.05).

**Table 7:** Effect of VTRS on activity of digestive enzymes in small intestinal tissue (n = 4).

Items	Groups		SEM	*p*-Value
	Control	VTRS		
Duodenum				
Amylase (U/g prot)	6.27 ^b^	16.31 ^a^	1.308	0.003
Lipase (U/mg prot)	6.29	7.10	1.302	0.683
Trypsin (U/mg prot)	34.48	32.75	2.810	0.678
Jejunum				
Amylase (U/g prot)	0.98	1.06	0.124	0.685
Lipase (U/mg prot)	14.44	13.88	2.145	0.860
Trypsin (U/mg prot)	143.7	146.2	17.39	0.922
Ileum				
Amylase (U/g prot)	1.90 ^b^	5.29 ^a^	0.506	0.010
Lipase (U/mg prot)	11.25	11.38	1.173	0.943
Trypsin (U/mg prot)	71.16	73.23	6.807	0.837

SEM—standard error of mean. Means within a row showing different superscripts were significant among treatments (*p* < 0.05).

**Table 8:** Effect of VTRS on VFA content in duodenal digesta (n = 4).

Items	Groups		SEM	*p*-Value
	Control	VTRS		
Concentration				
Acetate (mM)	1.55	2.27	0.248	0.091
Propionate (mM)	0.30	0.40	0.086	0.468
Butyrate (mM)	0.18 ^b^	1.96 ^a^	0.046	<0.001
Isobutyrate (mM)	0.17	0.18	0.003	0.061
Valerate (mM)	0.16	0.17	0.008	0.425
Isovalerate (mM)	0.18	0.23	0.016	0.112
^1^ A:P	6.75	7.17	2.422	0.907
^2^ TVFA (mmol/L)	2.54 ^b^	5.02 ^a^	0.207	<0.001
Molar proportions (%)				
Acetate	59.50	43.47	4.070	0.036
Propionate	11.97	7.76	2.375	0.264
Butyrate	7.46	37.62	1.345	<0.001
Isobutyrate	7.05	3.50	0.515	0.031
Valerate	6.68	3.32	0.525	0.024
Isovalerate	7.33	4.32	0.690	0.035

^1^ Acetate/Propionate; ^2^ Total volatile fatty acids; SEM—standard error of mean. Means within a row showing different superscripts were significant among treatments (*p* < 0.05).

**Table 9:** Effect of VTRS on VFA content in cecal digesta (n = 4).

Items	Groups		SEM	*p*-Value
	Control	VTRS		
Concentration				
Acetate (mM)	38.22 ^b^	42.98 ^a^	0.914	0.011
Propionate (mM)	12.86	14.03	1.054	0.462
Butyrate (mM)	4.87 ^b^	8.14 ^a^	0.532	0.005
Isobutyrate (mM)	1.12	1.31	0.088	0.242
Valerate (mM)	0.63 ^b^	1.27 ^a^	0.129	0.019
Isovalerate (mM)	0.56	0.60	0.077	0.692
^1^ A:P	3.02	3.11	0.195	0.761
^2^ TVFA (mM)	58.81 ^b^	70.61 ^a^	3.002	0.017
Molar proportions (%)				
Acetate	65.67	63.04	1.275	0.197
Propionate	21.97	20.45	1.011	0.329
Butyrate	8.37	11.86	0.675	0.014
Isobutyrate	1.92	1.93	0.185	0.996
Valerate	1.09	1.83	0.170	0.023
Isovalerate	0.95	0.88	0.101	0.623

^1^ Acetate/Propionate; ^2^ Total volatile fatty acids; SEM—standard error of mean. Means within a row showing different superscripts were significant among treatments (*p* < 0.05).

**Table 10:** Effect of VTRS on glucose and amino acid transporters in small intestinal tissue (n = 4).

Items	Groups		SEM	*p*-Value
	Control	Silage		
Duodenum				
^1^ GLUT2	1.00	1.17	0.304	0.673
^2^ SGLT1	1.00	1.02	0.406	0.898
^3^ SLC38A2	1.00	1.83	0.418	0.093
^4^ SLC3A2	1.00	0.84	0.169	0.065
^5^ SLC6A19	1.00	0.41	0.252	0.052
^6^ SLC1A5	1.00	0.62	0.203	0.135
Jejunum				
^1^ GLUT2	1.00	1.18	0.422	0.517
^2^ SGLT1	1.00	1.85	0.382	0.064
^3^ SLC38A2	1.00	0.87	0.239	0.620
^4^ SLC3A2	1.00	1.30	0.212	0.210
^5^ SLC6A19	1.00	1.02	0.348	0.936
^6^ SLC1A5	1.00	0.78	0.159	0.190
Ileum				
^1^ GLUT2	1.00 ^b^	5.73 ^a^	0.531	0.003
^2^ SGLT1	1.00 ^b^	1.98 ^a^	0.563	0.005
^3^ SLC38A2	1.00	1.42	0.398	0.137
^4^ SLC3A2	1.00	1.16	0.166	0.369
^5^ SLC6A19	1.00	1.52	0.418	0.245
^6^ SLC1A5	1.00	1.13	0.376	0.251

^1^ Glucose transporter 2; ^2^ Sodium–glucose cotransporter 1; ^3^ Amino acid transporter 2. ^4^ Solute carrier family 3 Member 2; ^5^ Solute carrier family 6 members 19; ^6^ Solute carrier family 1 member 5; SEM—standard error of mean; Means within a row showing different superscripts were significant among treatments (*p* < 0.05).

**Table 11:** Effect of VTRS on antioxidant status in seum and liver (n = 4).

Items	Groups			*p*-Value
	Control	VTRS	SEM	
Serum				
TAOC (U/mL)	0.38 ^b^	0.41 ^a^	0.006	0.003
SOD (U/mL)	253.0	263.5	13.05	0.720
GSH-Px (U/mL)	202.3	168.2	9.840	0.078
CAT (U/mgHb)	1.96	1.96	0.144	0.992
MDA (nmol/mL)	1.98 ^a^	1.57 ^b^	0.105	0.038
IL-1β (pg/mL)	483.6	490.6	24.10	0.897
IL-6 (pg/mL)	180.5	199.5	8.395	0.288
IL-8 (pg/mL)	14.89	14.58	0.834	0.869
IL-10 (pg/mL)	239.0 ^b^	391.0 ^a^	28.85	<0.001
TNF-α (pg/mL)	541.5	576.7	13.21	0.203
IgA (pg/mL)	33.74	46.49	4.427	0.163
IgM (pg/mL)	350.1	377.6	42.30	0.314
Liver				
TAOC (U/mL)	0.51	0.44	0.018	0.104
SOD (U/mL)	329.6	278.9	14.67	0.079
GSH-Px (U/mL)	179.8	184.8	8.203	0.788
CAT (U/mgHb)	0.94	0.72	0.085	0.226
MDA (nmol/mL)	1.39 ^a^	0.85 ^b^	0.120	0.007
IL-1β (pg/mL)	393.9	440.3	17.38	0.202
IL-6 (pg/mL)	398.6	404.5	17.06	0.879
IL-8 (pg/mL)	10.36	12.78	0.652	0.053
IL-10 (pg/mL)	336.5	367.8	12.17	0.222
TNF-α (pg/mL)	511.1	544.6	26.57	0.570
IgA (pg/mL)	40.07	48.31	3.338	0.244
IgM (pg/mL)	350.1	377.6	42.30	0.314

SEM—standard error of mean. Means within a row showing different superscripts were significant among treatments (*p* < 0.05).

**Table 12:** Effect of VTRS on antioxidant status in duodenum and caecum tissues (n = 4).

Items	Groups			*p*-Value
	Control	VTRS	SEM	
Duodenum				
TAOC (U/mL)	0.48 ^b^	0.51 ^a^	0.006	0.04
SOD (U/mL)	179.5	195.3	8.90	0.417
GSH-Px (U/mL)	181.5 ^b^	243.1 ^a^	11.90	<0.001
CAT (U/mgHb)	0.47 ^b^	0.56 ^a^	0.019	0.002
MDA(nmol/mL)	1.74	1.54	0.055	0.062
IL-1β (pg/mL)	606.1	654.6	22.31	0.311
IL-6 (pg/mL)	390.1 ^b^	473.2 ^a^	18.71	0.017
IL-8 (pg/mL)	5.65	6.01	0.108	0.089
IL-10 (pg/mL)	241.6	244.9	11.52	0.897
TNF-α (pg/mL)	422.4	356.1	25.79	0.222
IgA (pg/mL)	66.77 ^b^	92.73 ^a^	6.664	0.037
IgM (pg/mL)	621.7	766.1	36.59	0.314
Caecum				
TAOC (U/mL)	0.49 ^b^	0.95 ^a^	0.093	0.004
SOD (U/mL)	176.1	194.3	12.08	0.496
GSH-Px (U/mL)	150.5 ^b^	179.6 ^a^	6.393	0.006
CAT (U/mgHb)	1.92	2.27	0.178	0.328
MDA(nmol/mL)	2.72	2.31	0.122	0.092
IL-1β (pg/mL)	420.6	442.7	22.31	0.657
IL-6 (pg/mL)	230.8	257.2	12.09	0.314
IL-8 (pg/mL)	4.89	3.92	0.316	0.132
IL-10 (pg/mL)	165.3 ^b^	243.9 ^a^	17.77	0.018
TNF-α (pg/mL)	510.3	535.1	6.821	0.061
IgA (pg/mL)	60.17	68.71	3.686	0.278
IgM (pg/mL)	610.57	607.1	36.40	0.314

SEM—standard error of mean. Means within a row showing different superscripts were significant among treatments (*p* < 0.05).

## Data Availability

Data can be provided on reasonable request.

## Supplementary Materials

The following supporting information can be downloaded at https://www.mdpi.com/article/10.3390/antiox13121546/s1, Figure S1: Venn diagrams showing distribution of OTUs among dietary groups; Table S1: Feed Fungal alpha diversity indices differences in control and vegetable waste silage group; Table S2: Comparison of clean tags, effective tags and OTUs among treatments; Table S3: Effect of vegetable waste silage on alpha diversity in duodenum and caecum.

## References

[1] McGrath J., Duval S.M., Tamassia L.F.M., Kindermann M., Stemmler R.T., de Gouvea V.N., Acedo T.S., Immig I., Williams S.N., Celi P. (2018). Nutritional Strategies in Ruminants: A Lifetime Approach. Res. Vet. Sci..

[2] Areaya A.N. (2018). Major Non-Conventional Feed Resources of Fivestock. Int. J. Eng. Dev. Res..

[3] Guo Y. (2022). Mutant Resource for Chinese Cabbage. Nat. Food.

[4] Wang X., Dou Z., Shi X., Zou C., Liu D., Wang Z., Guan X., Sun Y., Wu G., Zhang B. (2021). Innovative Management Programme Reduces Environmental Impacts in Chinese Vegetable Production. Nat. Food.

[5] Liu Y., Wang T., Xing Z., Ma Y., Nan F., Pan L., Chen J. (2022). Anaerobic Co-Digestion of Chinese Cabbage Waste and Cow Manure at Mesophilic and Thermophilic Temperatures: Digestion Performance, Microbial Community, and Biogas Slurry Fertility. Bioresour. Technol..

[6] Solomon H.M., Kautter D.A., Lilly T., Rhodehamel E.J. (1990). Outgrowth of Clostridium Botulinum in Shredded Cabbage at Room Temperature under a Modified Atmosphere. J. Food Prot..

[7] Ngu N.T., Ledin I. (2005). Effects of Feeding Wastes from Brassica Species on Growth of Goats and Pesticide/Insecticide Residues in Goat Meat. Asian-Australas. J. Anim. Sci..

[8] Alengebawy A., Ran Y., Ghimire N., Osman A.I., Ai P. (2023). Rice Straw for Energy and Value-Added Products in China: A Review. Environ. Chem. Lett..

[9] Jackson M.G. (1977). Rice Straw as Livestock Feed. World Anim. Rev..

[10] Partovi E., Rouzbehan Y., Fazaeli H., Rezaei J. (2020). Broccoli Byproduct-Wheat Straw Silage as a Feed Resource for Fattening Lambs. Transl. Anim. Sci..

[11] Keshavarz V., Dehghan-Banadaky M., Ganjkhanlou M., Kazemi-Bonchenari M. (2023). Effects of Feeding Wheat Straw or Beet Pulp in Starters Supplemented with Either Soybean Oil or Palm Fatty Acids on Growth Performance and Urinary Purine Derivatives in Dairy Calves. Anim. Feed Sci. Technol..

[12] Han H., Zhang L., Shang Y., Wang M., Phillips C.J.C., Wang Y., Su C., Lian H., Fu T., Gao T. (2022). Replacement of Maize Silage and Soyabean Meal with Mulberry Silage in the Diet of Hu Lambs on Growth, Gastrointestinal Tissue Morphology, Rumen Fermentation Parameters and Microbial Diversity. Animals.

[13] Wang B., Luo H. (2021). Effects of Mulberry Leaf Silage on Antioxidant and Immunomodulatory Activity and Rumen Bacterial Community of Lambs. BMC Microbiol..

[14] Zhang X., Li C., Shahzad K., Han M., Guo Y., Huang X., Wu T., Wang L., Zhang Y., Tang H. (2022). Seasonal Differences in Fecal Microbial Community Structure and Metabolism of House-Feeding Chinese Merino Fine-Wool Sheep. Front. Vet. Sci..

[15] Xu X.-L., Zhao Y., Chen M.-M., Li Y., Li Y., Wu S.-J., Zhang J.-L., Zhang X.-S., Yu K., Lian Z.-X. (2023). Shifts in Intestinal Microbiota and Improvement of Sheep Immune Response to Resist Salmonella Infection Using Toll-like Receptor 4 (TLR4) Overexpression. Front. Microbiol..

[16] Hill D.A., Artis D. (2009). Intestinal Bacteria and the Regulation of Immune Cell Homeostasis. Annu. Rev. Immunol..

[17] Karakan T., Ozkul C., Küpeli Akkol E., Bilici S., Sobarzo-Sánchez E., Capasso R. (2021). Gut-Brain-Microbiota Axis: Antibiotics and Functional Gastrointestinal Disorders. Nutrients.

[18] Lozupone C.A., Stombaugh J.I., Gordon J.I., Jansson J.K., Knight R. (2012). Diversity, Stability and Resilience of the Human Gut Microbiota. Nature.

[19] Popova M., McGovern E., McCabe M.S., Martin C., Doreau M., Arbre M., Meale S.J., Morgavi D.P., Waters S.M. (2017). The Structural and Functional Capacity of Ruminal and Cecal Microbiota in Growing Cattle Was Unaffected by Dietary Supplementation of Linseed Oil and Nitrate. Front. Microbiol..

[20] de Oliveira M.N.V., Jewell K.A., Freitas F.S., Benjamin L.A., Tótola M.R., Borges A.C., Moraes C.A., Suen G. (2013). Characterizing the Microbiota across the Gastrointestinal Tract of a Brazilian Nelore Steer. Vet. Microbiol..

[21] Bergmann G.T. (2017). Microbial Community Composition along the Digestive Tract in Forage-and Grain-Fed Bison. BMC Vet. Res..

[22] Myer P.R., Wells J.E., Smith T.P., Kuehn L.A., Freetly H.C. (2015). Microbial Community Profiles of the Colon from Steers Differing in Feed Efficiency. Springerplus.

[23] Pan X.H., Yang L., Xue F.G., Xin H.R., Jiang L.S., Xiong B.H., Beckers Y. (2016). Relationship between Thiamine and Subacute Ruminal Acidosis Induced by a High-Grain Diet in Dairy Cows. J. Dairy Sci..

[24] Zhang Z., Shahzad K., Shen S., Dai R., Lu Y., Lu Z., Li C., Chen Y., Qi R., Gao P. (2022). Altering Dietary Soluble Protein Levels with Decreasing Crude Protein May Be a Potential Strategy to Improve Nitrogen Efficiency in Hu Sheep Based on Rumen Microbiome and Metabolomics. Front. Nutr..

[25] Zhang Z., Gu Y., Wang S., Zhen Y., Chen Y., Wang Y., Mao Y., Meng J., Duan Z., Xu J. (2024). Effective Microorganism Combinations Improve the Quality of Compost-Bedded Pack Products in Heifer Barns: Exploring Pack Bacteria-Fungi Interaction Mechanisms. BMC Microbiol..

[26] Li C., Chen N., Zhang X., Shahzad K., Qi R., Zhang Z., Lu Z., Lu Y., Yu X., Zafar M.H. (2022). Mixed Silage with Chinese Cabbage Waste Enhances Antioxidant Ability by Increasing Ascorbate and Aldarate Metabolism through Rumen Prevotellaceae UCG-004 in Hu Sheep. Front. Microbiol..

[27] Christensen C.M. (1982). Storage of Cereal Grains and Their Products.

[28] Umarov G.G., Abdurokhmonov S.X., Tulaganov B.Q., Telovov A.T., Bozorboev A.A. (2022). Washing Steps and the Process of Saturation of Contaminants with Moisture in the Processing of Fruits and Vegetables. Proceedings of the IOP Conference Series: Earth and Environmental Science.

[29] Rai A., Das M., Tripathi A. (2020). Occurrence and Toxicity of a Fusarium Mycotoxin, Zearalenone. Crit. Rev. Food Sci. Nutr..

[30] Zhang G.-L., Feng Y.-L., Song J.-L., Zhou X.-S. (2018). Zearalenone: A Mycotoxin with Different Toxic Effect in Domestic and Laboratory Animals’ Granulosa Cells. Front. Genet..

[31] Kaci H., Dombi Á., Gömbös P., Szabó A., Bakos É., Özvegy-Laczka C., Poór M. (2024). Interaction of Mycotoxins Zearalenone, α-Zearalenol, and β-Zearalenol with Cytochrome P450 (CYP1A2, 2C9, 2C19, 2D6, and 3A4) Enzymes and Organic Anion Transporting Polypeptides (OATP1A2, OATP1B1, OATP1B3, and OATP2B1). Toxicol. Vitr..

[32] Feng N., Zhong F., Cai G., Zheng W., Zou H., Gu J., Yuan Y., Zhu G., Liu Z., Bian J. (2023). Fusarium Mycotoxins Zearalenone and Deoxynivalenol Reduce Hepatocyte Innate Immune Response after the Listeria Monocytogenes Infection by Inhibiting the TLR2/NFκB Signaling Pathway. Int. J. Mol. Sci..

[33] Das N.G., Sultana N., Amanullah S.M., Jalil M.A., Huque K.S. (2022). Feeding of Vegetable Waste Silage to Lambs by Replacing Maize Silage. J. Appl. Anim. Res..

[34] Pokluda R. (2008). Nutritional Quality of Chinese Cabbage from Integrated Culture. Hortic. Sci..

[35] Patra A.K., Yu Z. (2022). Genomic Insights into the Distribution of Peptidases and Proteolytic Capacity among Prevotella and Paraprevotella Species. Microbiol. Spectr..

[36] Hasegawa T., Kakuta M., Yamaguchi R., Sato N., Mikami T., Murashita K., Nakaji S., Itoh K., Imoto S. (2022). Impact of Salivary and Pancreatic Amylase Gene Copy Numbers on Diabetes, Obesity, and Functional Profiles of Microbiome in Northern Japanese Population. Sci. Rep..

[37] Wang D., Tang G., Wang Y., Yu J., Chen L., Chen J., Wu Y., Zhang Y., Cao Y., Yao J. (2023). Rumen Bacterial Cluster Identification and Its Influence on Rumen Metabolites and Growth Performance of Young Goats. Anim. Nutr..

[38] Zhao F., He W., Wu T., Elmhadi M., Jiang N., Zhang A., Guan P. (2024). Supplementation of Coated Sodium Butyrate Relieved Weaning Stress and Reshaped Microbial Flora in Weaned Lambs. Front. Vet. Sci..

[39] Poulsen S.B., Fenton R.A., Rieg T. (2015). Sodium-Glucose Cotransport. Curr. Opin. Nephrol. Hypertens..

[40] Sun B., Chen H., Xue J., Li P., Fu X. (2023). The Role of GLUT2 in Glucose Metabolism in Multiple Organs and Tissues. Mol. Biol. Rep..

[41] Cui A., Huang T., Li S., Ma A., Pérez J.L., Sander C., Keskin D.B., Wu C.J., Fraenkel E., Hacohen N. (2024). Dictionary of Immune Responses to Cytokines at Single-Cell Resolution. Nature.

[42] Harvanová G., Duranková S., Bernasovská J. (2023). The Role of Cytokines and Chemokines in the Inflammatory Response. Alergol. Pol. J. Allergol..

[43] Arkhipov V.I., Pershina E.V., Levin S.G. (2019). The Role of Anti-Inflammatory Cytokines in Memory Processing in a Healthy Brain. Behav. Brain Res..

[44] Moore K.W., de Waal Malefyt R., Coffman R.L., O’Garra A. (2001). Interleukin-10 and the Interleukin-10 Receptor. Annu. Rev. Immunol..

[45] De Filippis F., Pasolli E., Ercolini D. (2020). Newly Explored Faecalibacterium Diversity Is Connected to Age, Lifestyle, Geography, and Disease. Curr. Biol..

[46] Lenoir M., Martín R., Torres-Maravilla E., Chadi S., González-Dávila P., Sokol H., Langella P., Chain F., Bermúdez-Humarán L.G. (2020). Butyrate Mediates Anti-Inflammatory Effects of Faecalibacterium Prausnitzii in Intestinal Epithelial Cells through Dact3. Gut Microbes.

[47] Ishida K., Kishi Y., Oishi K., Hirooka H., Kumagai H. (2015). Effects of Feeding Polyphenol-rich Winery Wastes on Digestibility, Nitrogen Utilization, Ruminal Fermentation, Antioxidant Status and Oxidative Stress in Wethers. Anim. Sci. J..

[48] Sena L.A., Chandel N.S. (2012). Physiological Roles of Mitochondrial Reactive Oxygen Species. Mol. Cell.

[49] Ko K.M., Leung H.Y. (2007). Enhancement of ATP Generation Capacity, Antioxidant Activity and Immunomodulatory Activities by Chinese Yang and Yin Tonifying Herbs. Chin. Med..

[50] Fu A., van Rooyen L., Evans L., Armstrong N., Avizonis D., Kin T., Bird G.H., Reddy A., Chouchani E.T., Liesa-Roig M. (2021). Glucose Metabolism and Pyruvate Carboxylase Enhance Glutathione Synthesis and Restrict Oxidative Stress in Pancreatic Islets. Cell Rep..

