# Supplementary material for: Duodenum and Caecum Microbial Shift Modulates Immune and Antioxidant Response Through Energy Homeostasis in Hu Sheep Fed Vegetable Waste and Rice Straw Silage
Source: Antioxidants (Basel). 2024 Dec 17;13(12):1546. doi: 10.3390/antiox13121546 (PMC11727283; doi:10.3390/antiox13121546)
Supplement: Supplementary file 1 [file antioxidants-13-01546-s001.zip › antioxidants-3338063-supplementary.pdf]

Table S1 Feed Fungal alpha diversity indices differences in control and VTRS group

Items	Control	VTRS	SEM	P-value
Observed species	205.67	240.00	9.65	0.059
Shannon	4.15	4.50	0.098	0.84
Simpson	0.90	0.88	0.011	0.41
Chao1	266.4^{b}	312.75^{a}	12.37	0.037
PD whole tree	44.75	48.43	2.31	0.49

SEM standard error of mean

Means within a row showing different superscripts were significant among treatments (P<0.05)

Table S2 Comparison of clean tags, effective tags and OTUs among treatments

Items	Groups		SEM	P-value
	Control	VTRS		
		Feed		
Clean tags	74639.3	64961.7	6325.03	0.275
Effective tags	74446.3	64710.3	6311.19	0.275
OTUs	253.67^{b}	282.00^{a}	9.558	0.043
		Duodenum		
Clean tags	58553.3	64563.3	4321.67	0.513
Effective tags	49251.3	55274	7459.64	0.827
OTUs	1487.3	1615.66	230.27	0.827
		Caecum		
Clean tags	67304.67	67838.33	1366.32	0.513
Effective tags	63341	59840.67	2679.63	0.513
OTUs	1145	1533	158.43	0.275

SEM standard error of mean

Means within a row showing different superscripts were significant among treatments (P<0.05)

Table S3 Effect of vegetable waste silage on alpha diversity in duodenum and caecum

Items	Groups		SEM	P-value
	Control	VTRS		
**Duodenum**				
Observed species	1346.3	1451.7	175.2	0.80
Shannon	6.90	6.93	0.12	0.92
Simpson	0.97	0.96	0.004	0.75
Chao1	1606.92	2511.16	397.8	0.30
**Caecum**				
Observed species	1067	1469.67	167.52	0.27
Shannon	5.86	6.56	0.48	0.53
Simpson	0.92	0.93	0.02	0.77
Chao1	1154.2	1685.7	221.02	0.27

SEM standard error of mean

Means within a row showing different superscripts were significant among treatments (P<0.05)

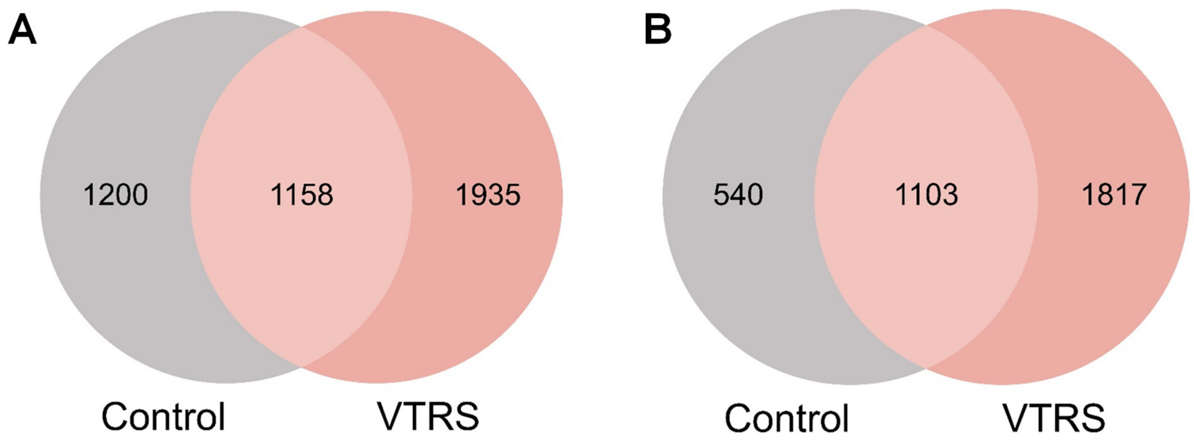

Figure S1 Venn diagram showing distribution of OTUs among dietary groups (A) Venn diagram for OUT distribution in duodenum (B) Venn diagram for OUT distribution in caecum
